# Fabrication of superhydrophobic and antibacterial surface on cotton fabric by doped silica**-**based sols with nanoparticles of copper

**DOI:** 10.1186/1556-276X-6-594

**Published:** 2011-11-15

**Authors:** Amirhosein Berendjchi, Ramin Khajavi, Mohammad Esmaeil Yazdanshenas

**Affiliations:** 1 Department of Textile Engineering, South Tehran Branch, Islamic Azad University, Tehran, Iran; 2 Department of Textile Engineering, Yazd Branch, Islamic Azad University, Yazd, Iran

**Keywords:** cotton, superhydrophobicity, antibacterial, sol-gel method, contact angle

## Abstract

The study discussed the synthesis of silica sol using the sol-gel method, doped with two different amounts of Cu nanoparticles. Cotton fabric samples were impregnated by the prepared sols and then dried and cured. To block hydroxyl groups, some samples were also treated with hexadecyltrimethoxysilane. The average particle size of colloidal silica nanoparticles were measured by the particle size analyzer. The morphology, roughness, and hydrophobic properties of the surface fabricated on cotton samples were analyzed and compared via the scanning electron microscopy, the transmission electron microscopy, the scanning probe microscopy, with static water contact angle (SWC), and water shedding angle measurements. Furthermore, the antibacterial efficiency of samples was quantitatively evaluated using AATCC 100 method. The addition of 0.5% (wt/wt) Cu into silica sol caused the silica nanoparticles to agglomerate in more grape-like clusters on cotton fabrics. Such fabricated surface revealed the highest value of SWC (155° for a 10-μl droplet) due to air trapping capability of its inclined structure. However, the presence of higher amounts of Cu nanoparticles (2% wt/wt) in silica sol resulted in the most slippery smooth surface on cotton fabrics. All fabricated surfaces containing Cu nanoparticles showed the perfect antibacterial activity against both of gram-negative and gram-positive bacteria.

## Background

Studying over 200 species of water repellent plants, Neinhuis and Barthlott [1] found an ideally wonderful superhydrophobic effect on lotus (*Nelumbo nucifera*) leaves which leads to supreme self-cleaning properties, so-called lotus effect [2,3]. The rough structure of lotus leaves (hills and valleys template) causes a reduced contact area with water. The presence of the hydrophobic nanoparticles, however, will prevent water from penetrating hills [4].

To simulate or produce such superhydrophobic surface on substrates, among different methods **(**such as chemical vapor deposition [5], phase inversion [6], electrospinning [7], electrowetting [8], lithography [9], and etching [10]**)**, the sol-gel method seems more conventional to be used on textile materials, due to easy processing and acceptable treatment conditions (e.g., low temperature) [11-17]. In this method, hydrolysis and condensation reactions of the precursor material are carried out to form a nano-colloidal solution, and a network of nanoparticles will be formed on the substrate through the gradual evaporation of the solvent. The precursors are often based on metal organic compounds such as acetylacetonate, or metal alkoxides like tetraethoxysilane Si(OC_2_H_5_)_4 _(TEOS), titanium(IV) isopropoxide Ti(OC_3_H_7_)_4_, and Al(OC_4_H_9_)_3 _[18].

According to its natural properties, cotton fabric is among the very popular textiles. Producing superhydrophobic surface on cotton fabric will guarantee its dryness and cleanness which are considered as desired features, in particular on its outside facet [11-17,19-21]. Furthermore, cotton fabric is an ideal place for settling and growing pathogenic bacteria because of its porous and hydrophilic structure. So, antibacterial finishing is also of importance, especially in some specific applications like medical usage. There are many antibacterial agents used in this field, including metal nanoparticles like silver and copper [22-29]. The latter is the most familiar antibacterial agent used for centuries. Like many other particles, the desired properties of copper may be improved by reducing its size to nano-scale. Hence, these nanoparticles can be developed and applied in various new fields, such as water purification, medical science, human tissue, antifouling and antibacterial agent, etc. [28].

Few researches have been focused on developing two abovementioned properties on cellulosic substrates like cotton fabric, simultaneously [30-32]. On the other hand, nanoparticles of copper and core shell SiO_2_/Cu have been less developed for textile finishing [33-36]. The current aimed to fabricate an antibacterial and superhydrophobic surface on the cotton fabric, by introducing Cu nanoparticles into the silica sols. It was expected that due to their chemical activities, such nanoparticles would change the morphology and arrangement of silica nanostructure, and in addition, promote antibacterial activity on cotton fabrics.

## Experimental

### Materials

Bleached and desized cotton fabric was provided by Polpine Co (Iran, Rasht). Tetraethylorthosilicate (TEOS), hydroxide ammonium (NH_4_OH 25%) and ethanol (C_2_H_5_OH 98%) were purchased from Merck Company. Nano-Cu (average particle size, 40 ±5 nm) was obtained from Plasma Company (PlasmaChem GmbH, Berlin, Germany), and hexadecyltrimethoxysilane (HDTMS) was purchased from Fluka Company (Sigma-Aldrich Chemie GmbH, Taufkirchen, Germany). All chemicals were used as received without any further purification.

### Methods

Colloidal SiO_2 _nanoparticle solutions were prepared considering the principles of Stöber method: 25 ml of ethanol, 1 ml of ammonium hydroxide, 3.6 ml of distilled water, and 11.5 ml of TEOS were mixed for 2 h at room temperature [37].

The prepared silica sols doped with two different amounts (0.5% and 2.0% wt/wt) of Cu nanoparticles, and were then sonicated for 30 min. Cotton fabric samples were immersed in the sols at 30°C for 5 min, dried for 24 h at ambient temperature and cured at 160°C for 5 min, respectively. Some samples were immersed in hydrolyzed and diluted HDTMS (with 4% wt/wt ethanol) for 4 h at room temperature. Again, these samples were cured at 120°C for 1 h [15].

### Characterization

Particle sizes of the silica sols prepared were measured with a particle size analyzer (Malvern Instruments, Malvern, Worcestershire, UK). The surface morphology was investigated by the scanning electron microscope (SEM) (XL30, Philips, Royal Philips Electronics, Amsterdam, Netherlands), while the surface roughness was analyzed via the scanning probe microscope (SPM) (DualScope™ C26, DME, Herlev, Denmark) using AC mode. The SiO_2_/Cu hybrid structure was observed with the transmission electron microscope (TEM) (EM 10C, Zeiss, Oberkochen, Germany). The static water contact angle (SWC) was determined by using a contact angle measurement device (Krüss G10, KRÜSS GmbH, Hamburg, Germany). At 23 ± 5°C, a 10-μl droplet of deionized water was placed into five different positions on the sample surfaces, and the angles of drops on the fabrics were determined. The static contact angle values for the sample reported were the average of five measurements.

Water shedding angle (WSA) of various samples was measured by the method of Zimmermann et al. [38]. After releasing a drop of water (15 μl) in a height of 1 cm, the minimum angle of inclination at which the drop completely rolls off the surface was determined.

The antibacterial activity of samples was quantitatively evaluated using AATCC 100 method. Two non-spore-forming bacteria, one Gram-positive *Staphylococcus aureus *(ATCC = 25923) and one Gram-negative *Escherichia coli *(ATCC = 25922), were used for antibacterial testing.

For determining the number of bacteria after 0 contact time, autoclaved swatches were placed in wide mouth glass jars and 100 μl of inoculums (containing 10^6 ^colony-forming units (CFU) was poured on each of them. Immediately after inoculation ("0" contact time), 100 ml of neutralizing solution (phosphate-buffered saline (PBS)) was added to each jar. After vigorous stirring (2,500 rpm for 1 min), the solution in each jar was poured on a nutrient agar plate.

For determining the number of bacteria after 24-h contact period, additional jars containing inoculated untreated control swatches and jars containing inoculated treated test swatches were incubated at 37°C for 24 h. The bacteria were eluted from each of the inoculated and incubated swatches by adding PBS (100 ml) neutralizing solution after vigorous stirring (2,500 rpm for 1 min). The solutions were poured on nutrient agar and all plates were incubated for further 18 h at 37°C. Finally, formed colony units were counted and antibacterial activity was reported in the percentage of reduction based on below equation (Equation 1):

(1) $$R\;=\text{ 1}00\left(B-A\right)∕B$$

where *R *is the percent reduction, *A *is the number of bacteria recovered from the inoculated treated test specimen swatches in the jar incubated over 24 h, and *B *is the number of bacteria recovered from the inoculated treated test specimen swatches in the jar immediately after inoculation (at "0" contact time).

## Results and discussions

**Figure 1:**
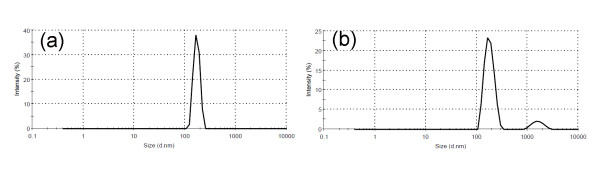
**Particle size analysis**. (**a**) Undoped sol. (**b**) 0.5% Cu just doped sol.

During the sol-gel process, TEOS was first hydrolyzed to silicic acid (Equation 2). Then, condensation reactions led to the formation of Si-O-Si bounds (Equation 3) and colloidal silica nanoparticles would be appeared by the emergence of a milky silica sol [37,39,40]. In this stage, synthesized nanoparticles of colloidal silica had the mean size of 80 nm (Figure 1a).

(2) $$\text{Si}\left(\text{O}{\text{C}}_{\text{2}}{\text{H}}_{\text{5}}\right)+{\text{H}}_{\text{2}}\text{O}⇄\text{Si}{\left(\text{OH}\right)}_{\text{4}}+\text{4}{\text{C}}_{\text{2}}{\text{H}}_{\text{5}}\text{OH}$$

(3) $$\text{2Si}{\left(\text{OH}\right)}_{4}\to \text{2}\left(\text{Si - O - Si}\right)\;+{\text{ 4H}}_{\text{2}}^{}\text{O}$$

**Figure 2:**
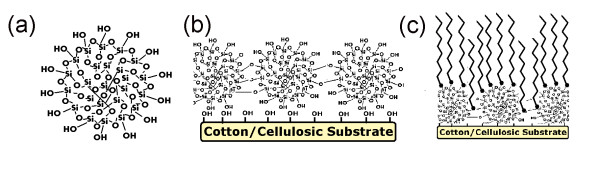
**Schematic drawings**. (**a**) A colloidal silica nanoparticle. (**b**) Silica nanostructured surface containing hydroxyl groups. (**c**) Silica substrate treated with alkylsilane.

**Figure 3:**
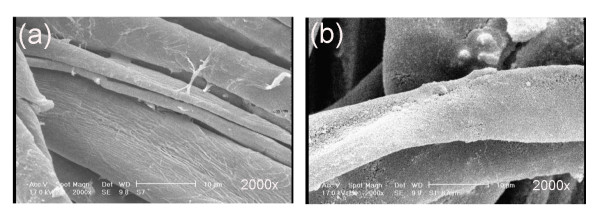
**SEM micrographs of different samples**. (**a**) Untreated. (**b**) Treated with SiO_2_.

After drying and curing, the solvent was evaporated and the agglomeration of silica nanoparticles fabricated silicon nanostructures on cotton fabrics. Since the presence of hydroxyl groups (Si-(OH)_3_) in silicon nanostructures, the surface fabricated still remained hydrophilic (Figure 2b). However, due to its covering effect on cotton fabrics, a water droplet could not easily penetrate into the fabric as in pristine fabric. The interaction of a long-chain alkylsilane agent like HDTMS with silanol groups produced a hydrophobic surface which in turn would increase the contact angle (Figure 2c). Figure 3 represented SEM images of untreated sample and cotton fabrics treated with alkylsilane and SiO_2 _nanoparticles. Alkylsilane-treated SiO_2 _surface (Figure 3b) apparently showed higher roughness than untreated one (Figure 3a). Its SWC and WSA were 151.1° and 30°, respectively (Table 1 Figure 3b). The contact angles of pristine fabric and samples only treated with alkylsilane or silica were not measurable, due to rapid absorbance of falling water droplets.

**Table 1:** Static water and water shedding angles of fabricated surface on cotton fabric samples

Kind of surface	Mean of static contact angle "SWC" (°)	Standard error of "SWC"	Mean of waster shedding angle "WSA" (°)	Standard error of "WSA"
SiO_2__Alylsilane	151.1	0.30	30	?
SiO_2__LowCu	155.9	0.64	24	?
SiO_2__HighCu	147.0	0.98	22	?

**Figure 4:**
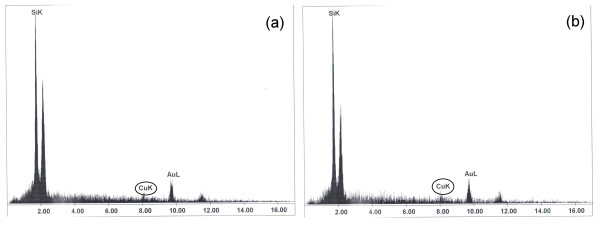
**EDX analysis for treated cotton fabrics containing different doped sols**. (**a**) 0.5% Cu. (**b**) 2% Cu.

When silica sol was doped with Cu nanoparticles and then cotton fabrics were immersed in it, as expected, the energy dispersive X-ray (EDX) analysis confirmed the presence of Cu nanoparticles on the sample surface (Figure 4).

**Figure 5:**
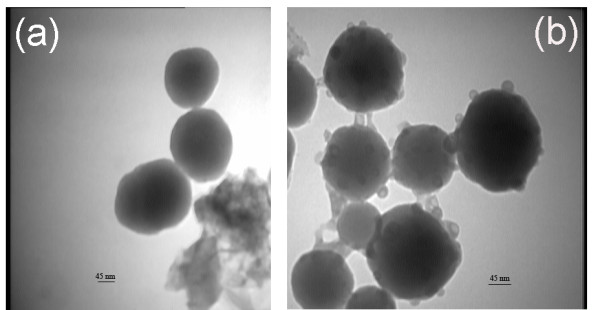
**TEM micrographs of the synthesized nanoparticles**. (**a**) Silica. (**b**) 0.5% Cu-doped silica.

Cu nanoparticles were introduced into the silica sols when the colloidal silica nanoparticles had been previously formed by the sol-gel process. Hence, they would be settled on the surface of colloidal SiO_2 _nanoparticles. This was confirmed by TEM images (Figure 5). Dissolution in alkaline silica sol may result to various cuprous and cupric complexes like Cu(OH)2, Cu_2_CO_3_(OH)_2_, Cu(NH_3_)^2+^, and Cu(NH3)_2_^+^, indicating a tendency towards colloidal silica nanoparticles.

The addition of 0.5% wt/wt Cu into silica sol caused the flocculation of colloidal silica nanoparticles (Figure 5b). The emersion of two peaks and the broadening of silica peaks in a size distribution graph just 5 min after introducing Cu nanoparticles may be attributed to the gradual agglomeration of silica and Cu particles (Figure 1b).

Such agglomeration would produce more grape-like clusters on the final fabricated surface. Compared with ordinary SiO_2 _nanostructured surface, this morphology showed higher air trapping capability and SWC(Table 1).

**Figure 6:**
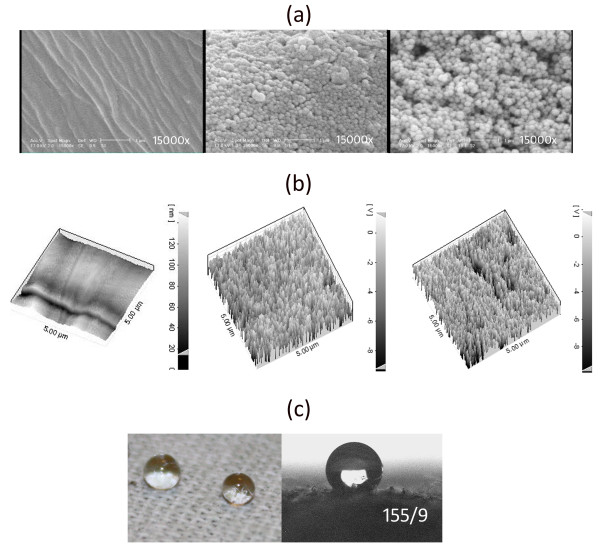
**SEM and SPM micrographs of fabricated surface**. Fabricated by: (**a **and **b**) (left) untreated, (middle) silica sol, (right) 0.5% Cu-doped silica, and (**c**) water drops on surface fabricated by 0.5% Cu-doped silica sol and its contact angle.

The valleys generated in (0.5%) Cu-doped treated samples were also obvious in SPM micrographs (Figure 6b).

Based on the fundamental theories on motion of liquid droplets on the rough surfaces [41], there are two important models about the wetting behavior of these surfaces: Wenzel and Cassie-Baxter [42]. The major difference between the two is the existence of air packets trapped in the valleys between liquid droplets and the solid substrates. Regarding the below equation [41,42], if the air pockets fraction (*f*_LA_) is high, then the value of cos*θ *is decreased which may be followed by the enhanced superhydrophobic effect on the roughened surfaces:

(4) $$\text{cos}\theta \;=\;R_{\text{f }}\text{cos}\theta_{0}-f_{\text{LA}}(R_{\text{f }}\text{cos}\theta_{0}+\text{1) }$$

where *R*_f _denotes the roughness factor, and *θ *and *θ*_0 _are the contact angles of liquid droplets on rough and flat surfaces, respectively. The WSA value for such sample was decreased and reached to 24°, and also, the slippery of treated surface was increased.

**Figure 7:**
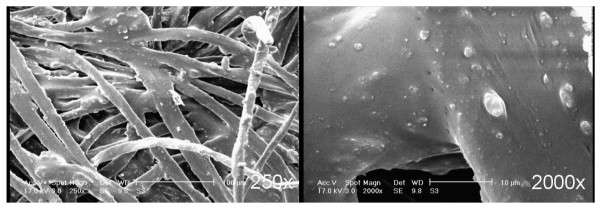
**SEM micrograph of 2% Cu-doped silica surface**.

**Figure 8:**
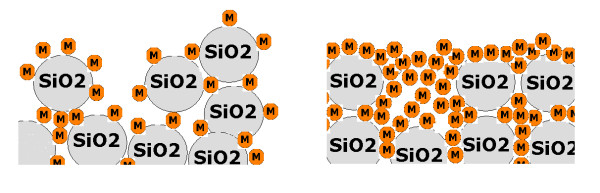
**Schematic drawings of the possible effect of Cu content on the morphology of fabricated surface**. Fabricated by: (left) 0.5% Cu-doped silica sol, (right) 2% Cu-doped silica sol.

Increasing the amount of Cu nanoparticles (2% owf) in silica sol may probably disintegrate the agglomerated clusters of silica nanoparticles and furthermore fill in the valleys of fabricated surfaces. Therefore, a homogeneous silica-copper hybrid nanocomposite would be formed on the cotton fabric samples (Figures 7 and 8). Creating a fairly flat level of roughness with filled-in valleys may result to a decreased SWC, comparing to silica networks with low Cu content (Table 1). In contrast with low Cu content silica network, however, a water droplet showed less tendency (or "petal effect") to adhere to surfaces of high Cu content silica network. This was consistent with WSA values, showing lower water shedding angle (Table 1).

Comparing data sets of static contacts angles "SWC" for different samples through analysis of variance demonstrated that differences between silica-alkylsilane, silica-Cu (0.5%) and silica-Cu (2%) were significantly important (significant value < 0.5). Standard errors for the measured static contact angles were increased by introducing Cu nanoparticles (Table 1). *Post hoc *test (Duncan's multiple range test) showed that (homogenous subsets of) means of all three abovementioned samples were different (Table 2). The effect of Cu nanoparticle on superhydrophobic property of the surface was even more than treating the surface with an alkylsilane agent like HDTMS. It should be noted that the silica-Cu (0.5%) can be considered as a hierarchical structure. In the case, the fabricated surface may have a self-cleaning capability, like *Hygroryza aristata *leaves.

**Table 2:** Post *post hoc *test (Duncan's multiple range test) for three samples

	Sample kind	*N*	Subset for alpha = 0.05		
			1	2	3
Duncan^a^	SiO2_Cu_2.0%	5	147.000		
	SiO2_AlkylSilane	5		151.140	
	SiO2_Cu_0.5%	5			155.900
	Significance		1.000	1.000	1.000

^a^Uses harmonic mean sample size = 5.000. Means for groups in homogeneous subsets are displayed.

All fabricated surface containing Cu nanoparticles displayed acceptable antibacterial properties against *E. coli *and *S. aureus *bacteria (Table 3). The criterion for passing the test or evaluating them was the percentage of bacteria growth reduction. Approximately, the total numbers of bacteria for samples were 22,800 (for *E. coli *bacteria) and 17,440 (for *S. aureus *bacteria) CFU/ml at zero contact time. These amounts reduced considerably (more than 70% for *E. coli *and 90% for *S. aureus *bacteria) for doped silica treated samples with Cu nano particles but increased for control samples (undoped silica treated). It may be attributed, first, to the antibacterial activities of Cu nanoparticles and, second, to the prohibition of Cu nanoparticles agglomeration resulted from their settlements on silica nanoparticles (Figure 5b). The latter is considered as an important parameter because it has been known that the antibacterial activity of metallic nanoparticles has a strong relationship with their sizes. The samples containing 2% Cu showed less antibacterial activity, especially against *E. coli *bacteria. This may result from the flocculation of Cu nanoparticles of high concentration.

**Table 3:** Percent reduction of bacteria on the fabricated control and doped silica surfaces

Sample kind	*Escherichia coli *(%)	*Staphylococcus aureus *(%)
	Gram negative	Gram positive
Silica-Cu (0.5%)	92.98	99.42
Silica-Cu (2%)	72.19	99.40
Undoped silica sol	0	0

## Conclusion

Copper, especially in its nano scale, has noticeable antibacterial activity with a more low cost compared with other similar antibacterial metals. In addition, the sol-gel method is a conventional process to coat thermo-sensitive substrates like cotton fabrics by nanoparticles. Introducing Cu nanoparticles into silica sol will fabricate a surface with higher air trapping capability on cotton fabrics. Therefore, it can imply superior properties of superhydrophobicity on the substrate and eliminate the need for post-treatment of silica surfaces with alkylsilane. Besides the intrinsic antibacterial properties, disintegration of Cu nanoparticle through the settling on SiO_2 _particles will simultaneously lead to an efficient antibacterial activity of the surface fabricated. Further study can also be conducted on more interesting properties such as self-cleaning capability of fabricated hierarchical surfaces.

## Competing interests

The authors declare that they have no competing interests.

## Authors' contributions

AB as a PhD student carried out experimental of the study and participated in its design, coordination and the sequence of alignments. RK as the super advisor of the project proposed the main idea and performed the experimental design of the study, interpretation of obtained data & its sequence alignment. MEY participated in its design and coordination as the consoler advisor of the project. All authors read & approved the final manuscript.

## References

[B1] Neinhuis C Barthlott W Characterization and distribution of water-repellent, self-cleaning plant surfaces Annals of Botany 1997 79 667 677 10.1006/anbo.1997.0400

[B2] Barthlott W Neinhuis C Purity of the sacred lotus, or escape from contamination in biological surfaces Planta 1997 202 1 8 10.1007/s004250050096

[B3] Mahltig B Textor T Improved water, oil and soil repellence Nanosols & Textiles 2008 USA: World Scientific 66 89

[B4] Hsieh CT Wu FL Yang SY Superhydrophobicity from composite nano/microstructures: Carbon fabrics coated with silica nanoparticles Surf Coat Tech 2008 202 6103 6108 10.1016/j.surfcoat.2008.07.006

[B5] Ma M Mao Y Gupta M Gleason KK Rutledge GC Superhydrophobic fabrics produced by electrospinning and chemical vapor deposition Macromolecules 2005 38 9742 9748 10.1021/ma0511189

[B6] Shi J Alves NM Mano JF Towards bioinspired superhydrophobic poly(L-lactic acid) surfaces using phase inversion-based methods Bioinspiration & Biomimetics 2008 3 1 6 10.1088/1748-3182/3/3/034003 18626131

[B7] Han D Steckl AJ Superhydrophobic and oleophobic fibers by coaxial electrospinning Langmuir 2009 25 9454 9462 10.1021/la900660v 19374456

[B8] Heikenfeld J Dhindsa M Electrowetting on superhydrophobic surfaces: present status and prospects J Adhesion Sci Tech 2008 22 319 334 10.1163/156856108X295347

[B9] Pozzato A Dal Zilio S Fois G Vendramin D Mistura G Belotti M Chen Y Natali M Superhydrophobic surfaces fabricated by nanoimprint lithography Microelectronic Engineering 2006 83 884 888 10.1016/j.mee.2006.01.012

[B10] Kim SH Kim JH Kang BK Uhm HS Superhydrophobic CF*x *coating via in-line atmospheric RF plasma of He-CF4-H2 Langmuir 2005 21 12213 12217 10.1021/la0521948 16342994

[B11] Bae GY Min BG Jeong YG Lee SC Jang JH Koo GH Superhydrophobicity of cotton fabrics treated with silica nanoparticles and water-repellent agent J Colloid Interface Sci 2009 337 170 175 10.1016/j.jcis.2009.04.066 19477460

[B12] Gao Q Zhu Q Guo Y Formation of highly hydrophobic surfaces on cotton and polyester fabrics using silica sol nanoparticles and nonfluorinated alkylsilane Ind Eng Chem Res 2009 48 9797 9803 10.1021/ie9005518

[B13] Hao LF An QF Xu W Wang QJ Synthesis of fluoro-containing superhydrophobic cotton fabric with washing resistant property using nano-SiO_2 _sol-gel method Adv Mater Res 2010 121-122 23 26

[B14] Li Z Xing Y Dai J Superhydrophobic surfaces prepared from water glass and non-fluorinated alkylsilane on cotton substrates Appl Surf Sci 2008 254 2131 2135 10.1016/j.apsusc.2007.08.083

[B15] Yu M Gu G Meng WD Qing FL Superhydrophobic cotton fabric coating based on a complex layer of silica nanoparticles and perfluorooctylated quaternary ammonium silane coupling agent Appl Surf Sci 2007 253 3669 3673 10.1016/j.apsusc.2006.07.086

[B16] Xue CH Jia ST Zhang J Tian LQ Superhydrophobic surfaces on cotton textiles by complex coating of silica nanoparticles and hydrophobization Thin Solid Films 2009 517 4593 4598 10.1016/j.tsf.2009.03.185

[B17] Xu B Cai Z Wang W Ge F Preparation of superhydrophobic cotton fabrics based on SiO2 nanoparticles and ZnO nanorod arrays with subsequent hydrophobic modification Surf Coat Technol 2010 204 1556 1561 10.1016/j.surfcoat.2009.09.086

[B18] Mahltig B Textor T Nanosol preparation and application Nanosols & Textiles 2008 USA: World Scientific 1 32

[B19] Erasmus E Barkhuysen FA Superhydrophobic cotton by fluorosilane modification Indian J Fibre & Textile Res 2009 34 377 379

[B20] Hoefnagels HF Wu D De With G Ming W Biomimetic superhydrophobic and highly oleophobic cotton textiles Langmuir 2007 23 13158 13163 10.1021/la702174x 17985939

[B21] Xu B Cai Z Fabrication of a superhydrophobic ZnO nanorod array film on cotton fabrics via a wet chemical route and hydrophobic modification Appl Surf Sci 2008 254 5899 5904 10.1016/j.apsusc.2008.03.160

[B22] Ravindra S Murali Mohan Y Narayana Reddy N Mohana Raju K Fabrication of antibacterial cotton fibers loaded with silver nanoparticles via "green approach" Colloids Surf A: Phys Eng Aspects 2010 367 31 40 10.1016/j.colsurfa.2010.06.013

[B23] Chen CY Chiang CL Preparation of cotton fibers with antibacterial silver nanoparticles Mater Letters 2008 62 3607 3609 10.1016/j.matlet.2008.04.008

[B24] Xu H Shi X Ma H Lv Y Zhang L Mao Z The preparation and antibacterial effects of dopa-cotton/AgNPs Appl Surf Sci 2011 257 6799 6803 10.1016/j.apsusc.2011.02.129

[B25] Hebeish A El-Shafei A Sharaf S Zaghloul S Novel precursors for green synthesis and application of silver nanoparticles in the realm of cotton finishing Carbohydrate Polymers 2011 84 605 613 10.1016/j.carbpol.2010.12.032

[B26] El-Rafie MH Mohamed AA Shaheen ThI Hebeish A Antimicrobial effect of silver nanoparticles produced by fungal process on cotton fabrics Carbohydrate Polymers 2010 80 779 782 10.1016/j.carbpol.2009.12.028

[B27] Perelshtein I Applerot G Perkas N Wehrschuetz-Sigl E Hasmann A Guebitz G Gedanken A CuO-cotton nanocomposite: formation, morphology and antibacterial activity Surf Coat Tech 2009 204 54 57 10.1016/j.surfcoat.2009.06.028

[B28] Grace M Chand N Bajpai SK Copper alginate-cotton cellulose (CACC) fibers with excellent antibacterial properties J Engineered Fibers and Fabrics 2009 4 24 35

[B29] Chattopadhyay DP Patel BH Effect of nanosized colloidal copper on cotton fabric J Engineered Fibers and Fabrics 2010 5 1 6

[B30] Shateri Khalil-Abad M Yazdanshenas ME Superhydrophobic antibacterial cotton textiles J Colloid Interface Sci 2010 351 293 298 10.1016/j.jcis.2010.07.049 20709327

[B31] Vilcnik A Jerman I Surca Vuk A Kozelj M Orel B Tomsic B Simoncic B Kovac J Structural properties and antibacterial effects of hydrophobic and oleophobic sol-gel coatings for cotton fabrics Langmuir 2009 25 5869 5880 10.1021/la803742c 19432495

[B32] Yang H Deng Y Preparation and physical properties of superhydrophobic papers J Colloid Interface Sci 2008 325 588 593 10.1016/j.jcis.2008.06.034 18603258

[B33] Kim YH Kim CW Cha HG Cha HJ Kang YC Kang YS Jo B Ahn GW Preparation and characterization of Cu-SiO_2 _nanocomposite Molecular Crystals and Liquid Crystals 2009 515 251 254 10.1080/15421400903479872

[B34] Cioffi N Torsi L Ditaranto N Tantillo G Ghibelli L Sabbatini L Bleve-Zacheo T D'Alessio M Zambonin PG Traversa E Copper nanoparticle/polymer composites with antifungal and bacteriostatic properties Chem Mater 2005 17 5255 5262 10.1021/cm0505244

[B35] Kim YH Lee DK Cha HG Kim CW Kang YC Kang YS Preparation and characterization of the antibacterial Cu nanoparticle formed on the surface of SiO_2 _nanoparticles J Phys Chem B 2006 110 24923 24928 10.1021/jp0656779 17149913

[B36] Zhang N Gao Y Zhang H Feng X Cai H Liu Y Preparation and characterization of core-shell structure of SiO2@Cu antibacterial agent Colloids Surf B Biointerfaces 2010 81 537 543 10.1016/j.colsurfb.2010.07.054 20729042

[B37] Stöber W Fink A Bohn E Controlled growth of mono disperse silica spheres in the micron size range J Colloid Interface Sci 1968 26 62 69 10.1016/0021-9797(68)90272-5

[B38] Zimmermann J Seeger S Reifler FA Water shedding angle: a new technique to evaluate the water-repellent properties of superhydrophobic surfaces Textile Research Journal 2009 79 1565 1570 10.1177/0040517509105074

[B39] Rao KS El-Hami K Kodaki T Matsushige K Makino K A novel method for synthesis of silica nanoparticles J Colloid Interface Sci 2005 289 125 131 10.1016/j.jcis.2005.02.019 15913636

[B40] Ibrahim IAM Zikry AAF Sharaf MA Preparation of spherical silica nanoparticles: Stober silica J American Sci 2010 6 985 989

[B41] Cassie ABD Baxter S Wettability of porous surfaces Trans Faraday Soc 1944 40 546 551

[B42] Nosonovsky M Bhushan B Superhydrophobic surfaces and emerging applications: non-adhesion, energy, green engineering Current Opinion in Colloid Interf Sci 2009 14 270 280 10.1016/j.cocis.2009.05.004

